# Preventive effects of mouthguard use while sleeping on recurrent aphthous stomatitis: Preliminary interventional study

**DOI:** 10.1002/cre2.88

**Published:** 2017-10-11

**Authors:** Hidesuke Tada, Natsumi Fujiwara, Takaaki Tsunematsu, Yoshiko Tada, Rieko Arakaki, Naofumi Tamaki, Naozumi Ishimaru, Yasusei Kudo

**Affiliations:** ^1^ Department of Oral Molecular Pathology Tokushima University Graduate School of Biomedical Sciences Japan; ^2^ Tada Dental Clinic Japan; ^3^ Department of Oral Healthcare Promotion Tokushima University Graduate School of Biomedical Sciences Japan; ^4^ Department of Pathology and Laboratory Medicine Tokushima University Graduate School of Biomedical Sciences Japan; ^5^ Department of Preventive Dentistry Tokushima University Graduate School of Biomedical Sciences Japan

**Keywords:** mouthguard, mucosal diseases, prevention, recurrent aphthous stomatitis

## Abstract

Recurrent aphthous stomatitis (RAS) is the most common inflammatory ulceration in the oral mucosa of otherwise healthy individuals and is often accompanied by severe pain. However, the etiology of RAS is not completely understood, and currently, no therapy can completely prevent RAS recurrence. In our clinical experience, we noticed that patients using a night guard, which is often used for bruxism treatment, did not develop RAS. Therefore, the aim of this study was to determine whether mouthguard use can suppress RAS development. The cohort of this interventional, prospective, single‐center, and self‐controlled study included 20 subjects who developed RAS at least once a month. The oral health of all the subjects was recorded for 60 days before and after intervention with a mouthguard. The average number of RAS incidences decreased from 5.5 to 1.0, the average days until healing decreased from 7.3 to 5.6, and the period with RAS decreased from 31.5 to 5.0 with mouthguard use. Mouthguard use may be beneficial for preventing RAS development.

## 1 INTRODUCTION

Recurrent aphthous stomatitis (RAS) is the most common inflammatory ulceration of the oral mucosa of otherwise healthy individuals and is characterized by a pseudomembrane surrounded by an erythematous halo, frequently observed in the oral mucosa of otherwise healthy individuals (Femiano et al., 2007). RAS lesions occur on nonkeratinized or poorly keratinized surface of the mucosa such as labial and buccal mucosa, maxillary and mandibular sulci, unattached gingiva, soft palate, tonsillar fauces, floor of the mouth, ventral surface of the tongue, and inferior lateral surface of the tongue (Baccaglini et al., 2011; Femiano, Buonaiuto, Gombos, Lanza, & Cirillo, 2010). RAS manifests in the form of outbreaks with a chronic and self‐limiting course in most cases (Chavan et al., 2012; Preeti, Magesh, Rajkumar, & Karthik, 2011). It occurs in approximately 20% of the general population, although the incidence varies from 5% to 50% depending on the ethnic and socioeconomic groups studied (Chavan et al., 2012; Meng et al., 2009; Preeti et al., 2011; Quijano & Rodríguez, 2008; Zhou et al., 2010). Patients with RAS often complain of severe pain. Although multiple factors including genetic factors, food allergens, local trauma, endocrine alterations (menstrual cycle), stress and anxiety, smoking cessation, certain chemical products, and microbial agents are known to affect to the appearance of oral aphthae, the underlying etiology of RAS is not completely understood (Chavan et al., 2012; Femiano et al., 2010; Preeti et al., 2011; Volkov et al., 2009; Zhou et al., 2010).

A mechanism through cellular immune response has been proposed in the immunopathogenesis of RAS (Beguerie et al., 2015). Although the immunopathogenesis of RAS remains to be fully elucidated, T‐lymphocyte infiltration of the epithelium is likely to be in response to antigenic stimulation of oral mucosal keratinocytes. Keratinocyte death is mediated by cytotoxic T cells and involves the production of pro‐inflammatory cytokines such as interleukin (IL)‐2 and tumor necrosis factor (TNF)‐α (Natah et al., 2000). TNF‐α induces inflammation via endothelial cell adhesion and neutrophil chemotaxis (Natah et al., 2000); thus, it plays an important role in the development of new RAS lesions and has been found to be increased by twofold to fivefold in the saliva of affected patients (Eguia‐del Valle et al., 2011). Changes in elements of the salivary defense system, such as the enzyme superoxide dismutase levels, may be involved in the inflammatory response of RAS (Momen‐Beitollahi et al., 2010). Furthermore, many systemic diseases are known to be associated with aphthous‐like ulcers, including Behçet's disease; hematological disorders; vitamin deficiencies; gastrointestinal diseases; cyclic neutropenia; Reiter syndrome; mouth and genital ulcers with inflamed cartilage syndrome; periodic fever, aphthous stomatitis, pharyngitis, and cervical adenitis; Sweet's syndrome; and immune deficiencies (Baccaglini et al., 2011; Lalla et al., 2012). The diagnosis of RAS is based on anamnesis and clinical manifestations. Although there is no specific diagnostic test, it is essential to discard possible underlying systemic causes, particularly in adults with sudden outbreaks of RAS. Therapy for RAS is aimed to reduce the pain, promote healing, and decrease the frequency of outbreaks as much as possible. Clinical management of RAS includes the use of topical corticosteroids in the form of paste, a patch, or a pellet. Laser irradiation, anesthetic spray, and antiseptic mouthwash are also recommended to reduce the pain. However, currently, no therapy can completely prevent RAS recurrence.

A night guard is most often used as a treatment for bruxism to protect the teeth from hyperocclusal forces. A common definition of a night guard is an acrylic splint, either maxillary or mandibular, that assists the condyles in reaching their most anterior–superior position in the fossa (Dawson, 1989). There are many types of intraoral devices that vary by application, shapes, thickness, and constituting materials, which include ethylene‐vinyl acetate (EVA) copolymer, polymethylmethacrylate, and polyethylene terephthalate. An intraoral device may be referred to as a mouth guard, mouth protector, mouth piece, or gum shield when used for contact sports; as an occlusal splint, bite splint, or bite plane for the treatment of a temporomandibular disorder; and as a mouth tray when used for tooth bleaching and orthodontic therapy. In our clinical experience, we noticed that patients who used as a night guard while sleeping did not develop RAS. On the basis of our clinical experience, we hypothesized that the mouthguard use while sleeping may prevent RAS development. To demonstrate this, we performed a clinical trial of using a mouthguard for preventing RAS development. This is the first trial for preventing RAS by using a mouthguard.

## 2 SUBJECTS AND METHODS

### 2.1 Design

The protocol of this interventional, prospective, single‐center, self‐controlled study was approved by the Ethics Committee of the Tokushima University Graduate School of Biomedical Sciences (approval no. 2053), and the study was conducted in accordance with the tenets of the Declaration of Helsinki.

### 2.2 Study population

The study cohort included 27 patients with RAS who met the diagnostic criteria described by Femiano et al. (2007), with at least one recurrence of ulceration every month. The patients were treated at a private dental office under the administration of the Tokushima University. All subjects were diagnosed with a combination of minor and major RAS, but no other oral mucosal disease. Subjects with an ulceration associated with the following criteria were excluded:

- systemic diseases,
- alcohol abusers,
- patients with abnormal hematological screening results,
- patients with administered systemic or topical corticosteroid therapy,
- patients with an immune‐modulating treatment,
- pregnant, and
- breastfeeding woman.

**Figure 1:**
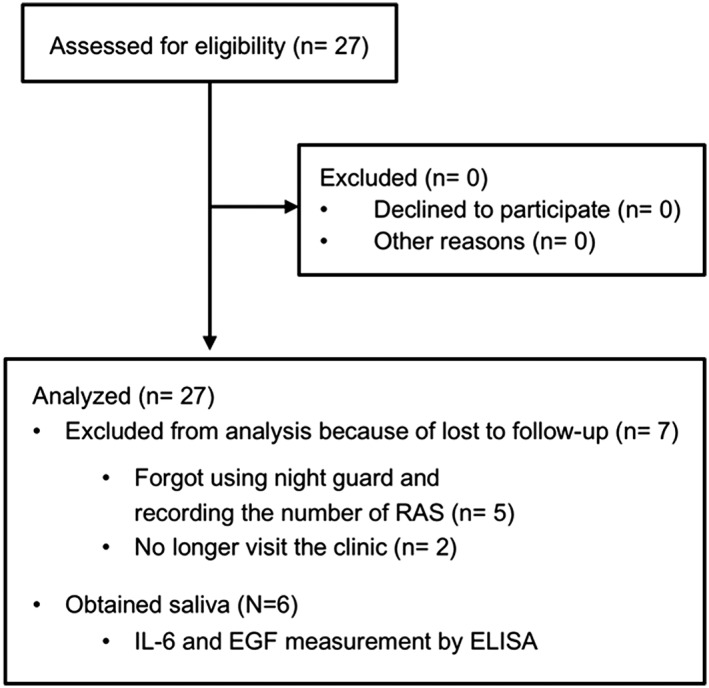
A flow diagram of the study protocol. EGF = epidermal growth factor; IL‐6 = interleukin‐6; RAS = recurrent aphthous stomatitis

Among the 27 subjects, seven were lost to follow‐up; thus, 20 subjects (12 females and 8 males; median age, 41.5 years) were included for analysis (Figure 1). For analysis, we considered the parameters as the following:

- number of RAS lesions,
- days until healing, and
- RAS duration period per 60 days.

During clinical trial, some subjects used antihypertensive drug, hypocholesterolemic drug, hypoglycemic drug, and headache medication. However, no subject used steroid, antimicrobials, vitamin B, and anti‐inflammatory agents. Therefore, all subjects did not take any drugs that may have been contributed to the healing.

### 2.3 Intervention

**Figure 2:**
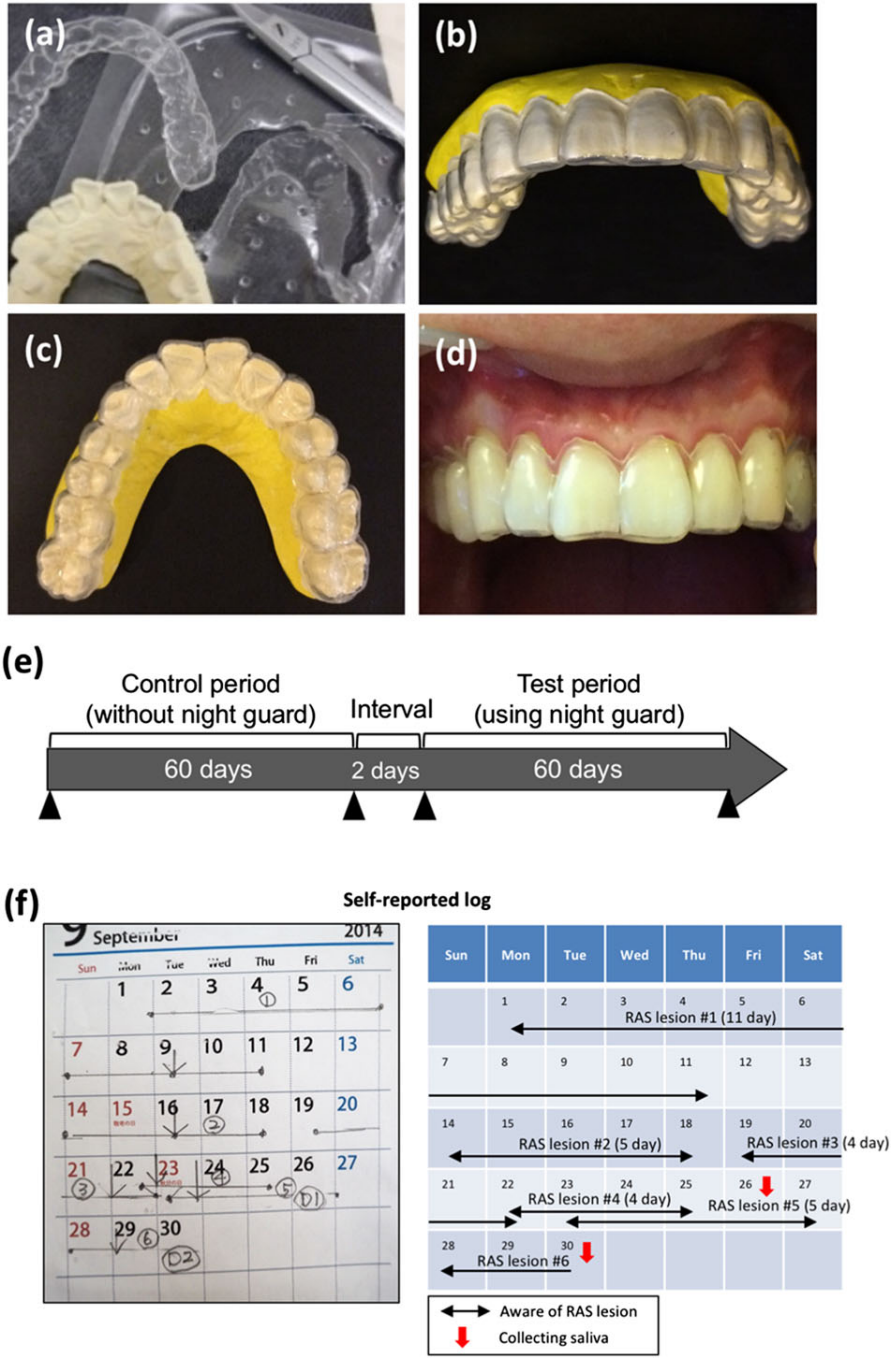
The mouthguard used in this study. (a) The mouthguard was fabricated using a 1.0‐mm‐thick ethylene‐vinyl acetate sheet according to the manufacturer's recommendations. The mouthguard was trimmed along the gingival margin using scissors. (b) Shape of the mouthguard. (c and d) Images during fitting of mouthguard in the oral cavity. All subjects used mouthguard only while sleeping, immediately removed the device upon awaking, and washed it with denture cleaner. (e) Schedule of this study. Subjects entered a 2‐day washout period, followed by a 60‐day control period and a second 60‐day period with mouthguard use while sleeping. RAS = recurrent aphthous stomatitis

In our clinical experience, we noticed that patients who used as a night guard while sleeping did not develop RAS. As it is difficult to use a night guard for subjects without bruxism or temporomandibular disorder, we used a soft and thinner type of mouthguard in this clinical trial. To ensure the comfort of the study subjects when wearing the mouthguard while sleeping during the testing period, a thinner type of mouthguard was desirable. In order to minimize the number of subjects discontinuing the mouthguard use as much as possible, a 1.0‐mm‐thick, soft, thermoplastic, EVA sheet (Mouth guard®, Yamahachi Dental Mfg. Co., Aichi, Japan) was used to fabricate each device in accordance with the manufacture's recommendations. EVA sheets are often used by dentists to fabricate sports guards, mouth trays for tooth whitening, and night guards. The safety of EVA has been recognized by the U.S. Food and Drug Administration (Code of Federal Regulations Title 21, Section 177.1350). In this clinical trial, in order to prevent discomfort and occlusal interference in subjects, we used a very thin (1.0 mm thick) EVA sheet. Indeed, mouthguard used in this study did not protect teeth and did not change the occlusion into more favorable and balanced position. Therefore, a very thin EVA sheet is not required for occlusal adjustment. For this study, all mouthguards were fabricated using a vacuum former (Vacuum Adaptor Type I, Yamahachi Dental Mfg. Co., Aichi, Japan) according to the manufacturer's recommendation using an impression of the maxillary teeth. The mouthguard was trimmed along the gingival margin to cover the dental arch without preventing saliva secretion (Figure 2a–d).

Each subject included in this trial underwent a general evaluation to arrive at a clinical diagnosis and submitted an informed consent form. The subjects underwent a 2‐day interval followed by a 60‐day control period without mouthguard use while sleeping. Then, the subjects entered a second 60‐day period with mouthguard use while sleeping (Figure 2e).

Treatment was considered effective if there was a reduction in the recovery period and frequency of RAS. All subjects were required to complete a self‐reported log regarding actual signs and symptoms of RAS every day (Figure 2f). Using these data, the duration and frequency of RAS were calculated.

### 2.4 Statistical analysis

The power of the study was calculated. The number of RAS‐positive days was chosen for the sample size calculation, and 14 days was accepted as the minimum clinically significant difference between before and after intervention in a preliminary study. A sample size of 18 patients was required for detection of a minimum clinically significant difference based on the power calculation of 90% power with a 5% Type I error level. A total of 27 subjects were targeted in this study to compensate for potential dropouts. Continuous variables and categorical data are expressed as medians (25th and 75th percentiles), and the number of subjects is expressed as a percentage. Because the data were skewed, clinical and biochemical parameters were analyzed using the Wilcoxon signed‐rank tests. JMP software program (version 12, SAS Institute, Cary, NC, USA) was used for all statistical analyses with a *p* value >.05 considered as significant.

## 3 RESULTS

Of the 27 subjects, seven (26%) were lost to follow‐up (Figure 1). The baseline demographic variables and risk factors associated with RAS of the 20 subjects who completed the study protocol are shown in Table 1. To determine the efficacy of mouthguard use for prevention of RAS development, in this clinical trial, we included 20 subjects who experienced RAS at least once per month. A clinical trial, as opposed to a randomized control trial, was performed because it was difficult to include a placebo group. As shown in Figure 2e, after a 2‐day interval and a 60‐day control period, a mouthguard was used while sleeping for 60 days. Twenty subjects had RAS in their oral cavities for 31.5 of 60 days before intervention (average score, 31.5 days/60 days). After intervention, the average score decreased to 5.0 days/60 days (Table 2). Mouthguard use significantly reduced the period with RAS (*p* < .0001, Table 2) and the number of RAS lesions (from 5.5 to 2.0, *p* < .0001, Table 2). Days until healing also significantly shortened from 7.3 to 5.6 days (*p* = .0007, Table 2). As shown in Figure S1a–c, all subjects showed an improvement in RAS incidence, days until healing, and the period with RAS. The use of prosthetic appliances (i.e., crowns, resin fillings, and partial dentures) did not influence the prevention of RAS development by mouthguard use.

**Table 1:** Clinical characteristics of the present subjects

Age (years, median [25%, 75%])	41.5 (33.8, 61.0)
Gender (male, *n* [%])	8 (40)
Number of teeth present (median [25%, 75%])	28 (24, 30)
Number of filling teeth (median [25%, 75%])	14 (8.8, 18.3)
Denture (yes, *n* [%])	1 (5)
Dental bridge (yes, *n* [%])	6 (30)
Tobacco exposure (yes, *n* [%])	2 (10)
Diabetes mellitus (yes, *n* [%])	1 (5)
Hypertension (yes, *n* [%])	2 (10)
Hypercholesterolemia (yes, *n* [%])	2 (10)
Articular rheumatism (yes, *n* [%])	1 (5)
Chronic rhinitis (yes, *n* [%])	2 (10)
Migraine headache (yes, *n* [%])	1 (5)

**Table 2:** Effects of using the mouthguard against RAS status

	Intervention (mouthguard)		*p* a
	−	+	
Number of RAS	5.5 (2.0, 8.3)	1.0 (0, 3.0)	<.0001
Days until healing	7.3 (3.3, 8.7)	5.6 (2.5, 7.5)	.0007
RAS duration period per 60 days (days)	31.5 (13.0, 41.0)	5.0 (0, 14.3)	<.0001

*Note*. Data are expressed as the median (25%, 75%). RAS = recurrent aphthous stomatitis.

a Wilcoxon signed‐rank test.

A mechanism through cellular immune response has been proposed in the immunopathogenesis of RAS (Beguerie et al., 2015). Trigger factors, which may include viral and bacterial antigens or stress, may initiate the cascade of pro‐inflammatory cytokines against the oral mucosa resulting in activation of T lymphocytes and leukocyte chemotaxis (Ślebioda, Szponar, & Kowalska, 2014). To investigate changes in cytokine/chemokine levels in saliva before and after intervention in a preliminary experiment, saliva samples from one of subject were collected during control and testing periods apart from this study (Figure S2a). Expression levels of epidermal growth factor (EGF) and IL‐6 increased during the period with RAS without mouthguard use, but decreased with mouthguard use (Figure S2b). Of the 20 subjects, saliva was obtained from six during the control and testing periods. Interestingly, IL‐6 secretion in saliva decreased in five of the six subjects during mouthguard use compared with during the control period (Figure S3a and Table S1), although the difference was not significant (*p* = .0781). EGF secretion was decreased during mouthguard use in only three of the six subjects (Figure S3b and Table S1).

## 4 DISCUSSION

RAS is reportedly the most common inflammatory ulcerative condition of the oral cavity (Shulman, 2004). As it is often accompanied by severe pain, the goal of RAS treatment is to reduce pain and healing time for restoring the ability to eat, swallow, and talk and improving the quality of life. Although the underlying etiology of RAS is not completely understood, several local, systemic, immunological, genetic, allergic, nutritional, and microbial factors have been proposed as causative agents (Akintoye & Greenberg, 2005). There is a huge range of supposed or possible remedies available, but objective evidence shows the most efficacy from corticosteroids and antimicrobials used topically (Baccaglini et al., 2011; Brocklehurst et al., 2012). However, currently, there is no definitive curative treatment for RAS. In this study, we performed a clinical trial for prevention of RAS development using a mouthguard while sleeping. Indeed, RAS development was effectively prevented, and healing was accelerated with mouthguard use (Table 2 and Figure S1). The advantages of using a mouthguard made from a very thin EVA sheet for the prevention of RAS include the relatively simple fabrication process (which requires no special technical skill), low material cost, safety, and simple maintenance.

To date, most studies aimed at determining the cause of RAS have focused on the detection of abnormal immunological responses. Correlation between RAS development and several immune‐mediated reactions, including cytotoxicity of T lymphocytes to the oral epithelium, antibody‐dependent cytotoxicity, and defects in lymphocyte subpopulations, have been suggested (Greenspan et al., 1985; Hoover, Olson, & Greenspan, 1986; Savage, Seymour, & Kruger, 1985). A recent study reported that physiological mechanical damage through mastication, via induction of IL‐6 from epithelial cells, tailored effector T‐cell function and promoted an increase in the cell numbers of gingival T helper 17 cells, which are key mediators of barrier immunity (Dutzan et al., 2017). Interestingly, barrier damage triggers oral T helper 17 cell‐mediated protective immunity and inflammation. Our preliminary comprehensive analysis of cytokine/chemokine levels in saliva during the period with or without intervention showed that expression levels of IL‐6 and EGF, which reflect the state of RAS, were reduced after mouthguard use (Figure S2b). Although TNF‐α is believed to play an important role in the development of new RAS lesions and has been found to be increased by twofold to fivefold in the saliva of affected patients (Eguia‐del Valle et al., 2011), we could not find a possible correlation of TNF‐α secretion with mouthguard use and RAS development. Indeed, downregulation of IL‐6, but not EGF, was correlated with the status of mouthguard use (Figure S3a and S3b). A mouthguard used in this study covers gingival sulcus. Therefore, we suggest that mouthguard may physically block the exposure of IL‐6, secreted from gingiva and gingival sulcus, to oral cavity via physiological mechanical damage through mastication while sleeping. Previous study show that serum IL‐6 level is frequently elevated in RAS patients (Yamamoto, Yoneda, Ueta, & Osaki, 1994). Moreover, inheritance of −174 promoter polymorphism of *IL‐6* is thought to be a strong predictor of RAS (Bazrafshani, Hajeer, Ollier, & Thornhill, 2002; Najafi et al., 2015), indicating that the elevated IL‐6 level in saliva may be involved in RAS development. To demonstrate this hypothesis, further experiments will be required.

Here, mouthguard use dramatically suppressed the RAS development and shortened the healing period without any side effects in 20 subjects. However, present clinical trial has been done within the limitations of this study regarding a 2‐month testing period in a single‐center, a self‐reported log of actual signs and symptoms of RAS. In addition, we did not consider the severity score of RAS and the effects of physiological conditions including nutrition status and stress in subjects. Therefore, future clinical trials with large numbers of subjects are needed to confirm these results. Moreover, it is necessary to clarify the mechanism of RAS prevention with mouthguard use. Overall, the results of this study showed that mouthguard use while sleeping is beneficial for preventing RAS development. Mouthguard use may be beneficial for preventing RAS development.

## CONFLICT OF INTEREST

The authors declare no potential conflict of interests with respect to the authorship and/or publication of this manuscript.

## Supporting information

Table S1. Supporting info item

Figure S1. Supporting info item

Figure S2. Supporting info item

Figure S2. Supporting info item

Figure S3. Supporting info item
