# Supplementary figures and images for: Preventive effects of mouthguard use while sleeping on recurrent aphthous stomatitis: Preliminary interventional study
Source: Clin Exp Dent Res. 2017 Oct 11;3(5):198–203. doi: 10.1002/cre2.88 (PMC5839199; doi:10.1002/cre2.88)

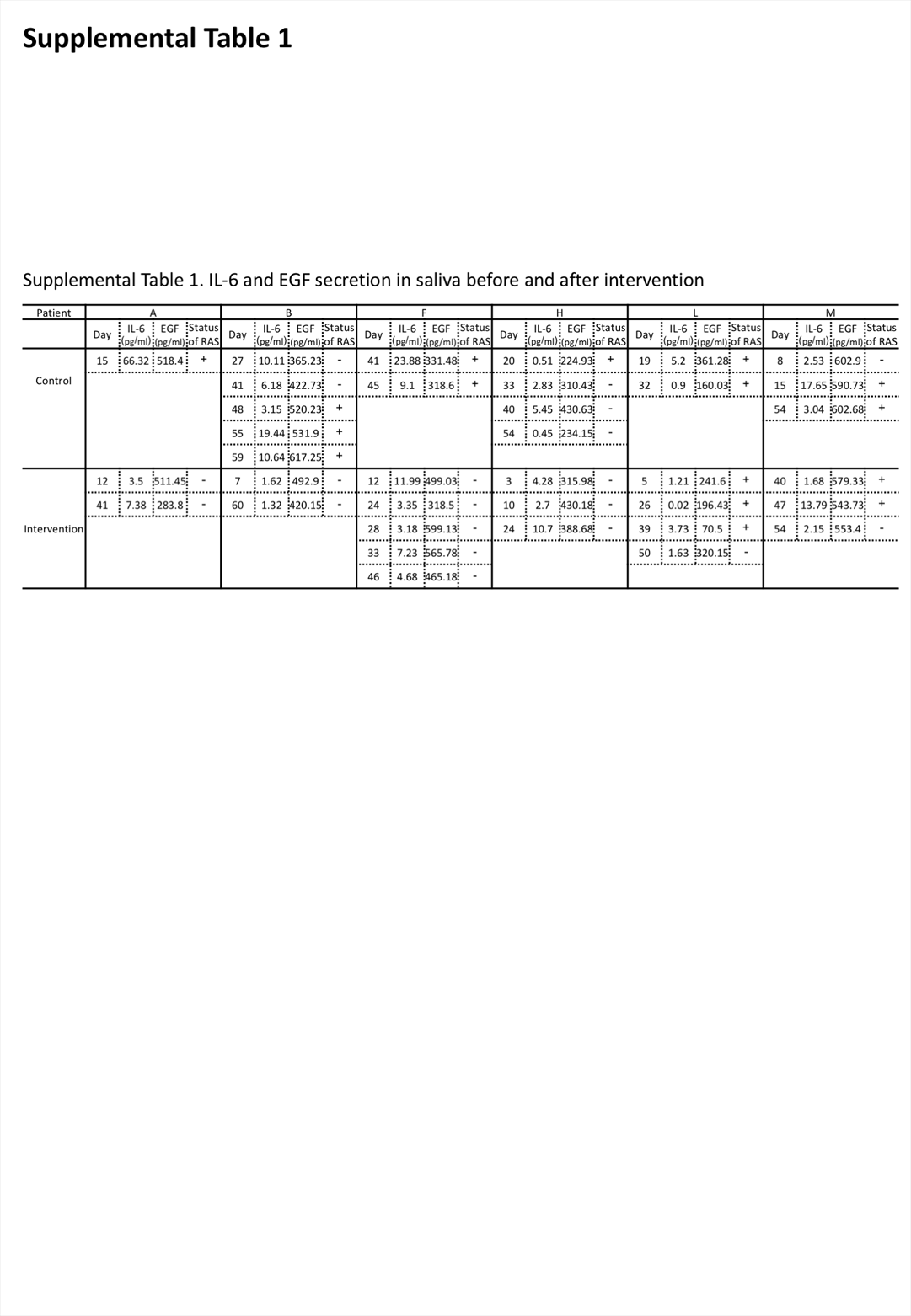

Supplement: Supplementary file 1 — Table S1. Supporting info item [file CRE2-3-198-s001.tiff]

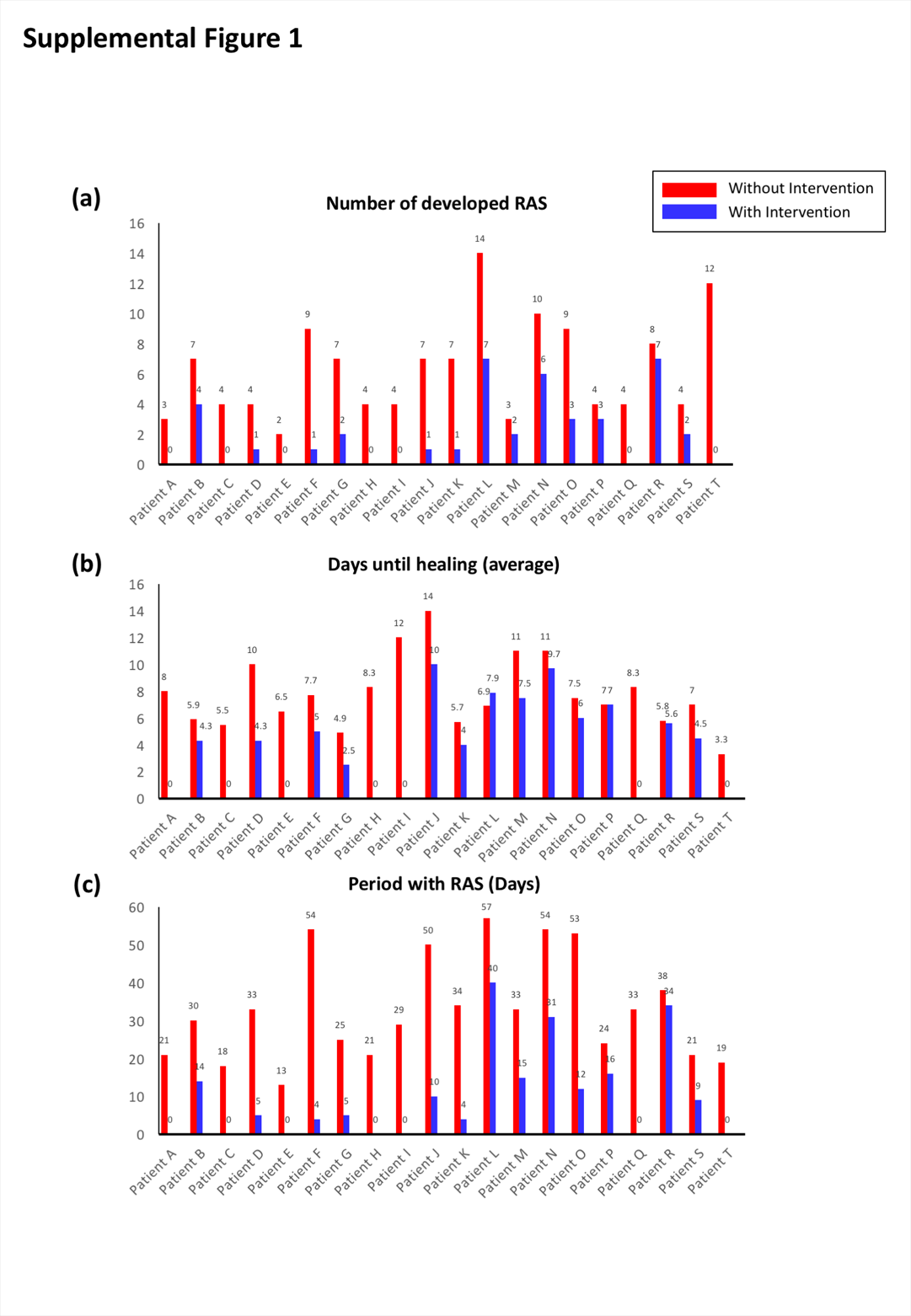

Supplement: Supplementary file 2 — Figure S1. Supporting info item [file CRE2-3-198-s002.tif]

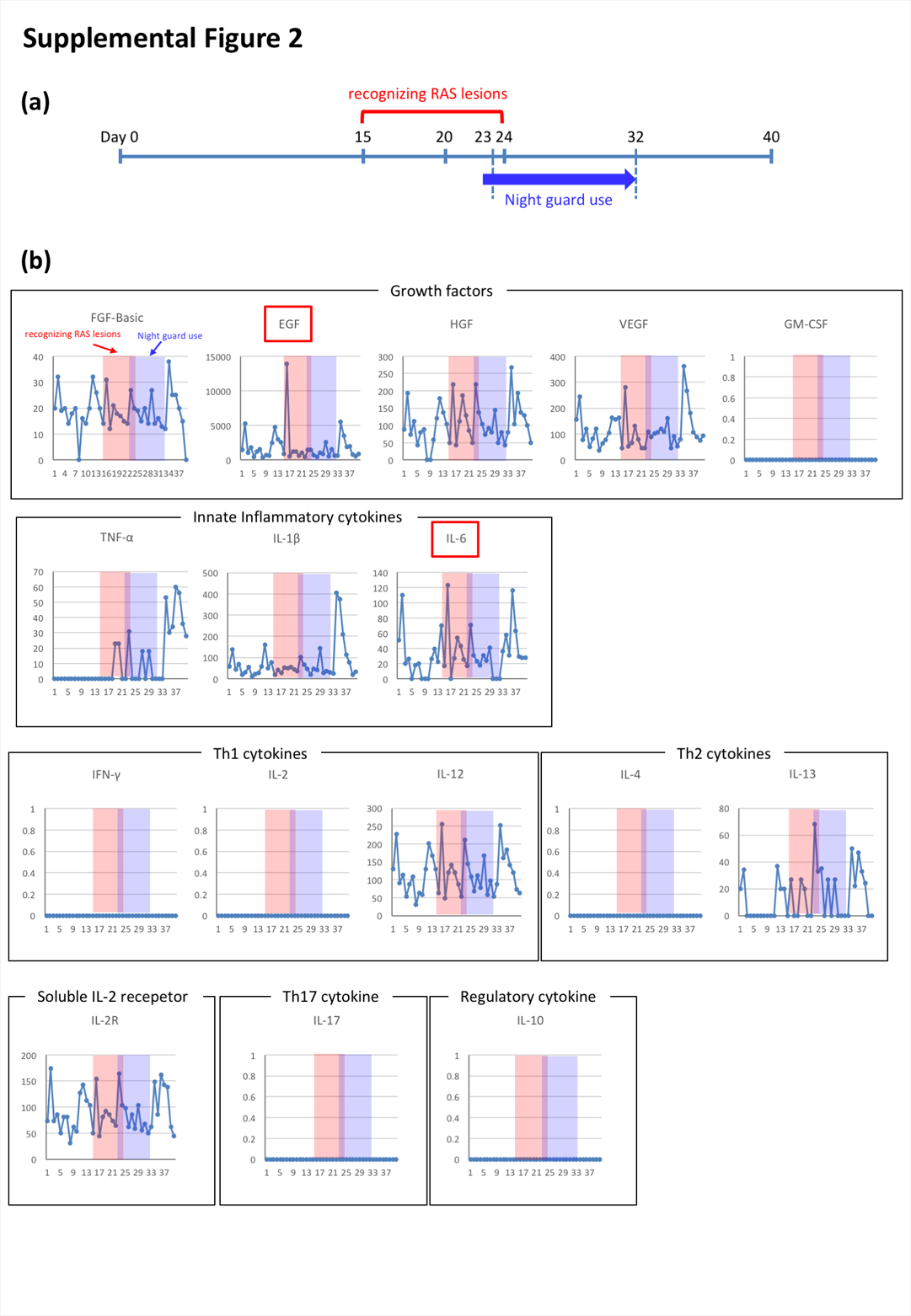

Supplement: Supplementary file 3 — Figure S2. Supporting info item [file CRE2-3-198-s003.tif]

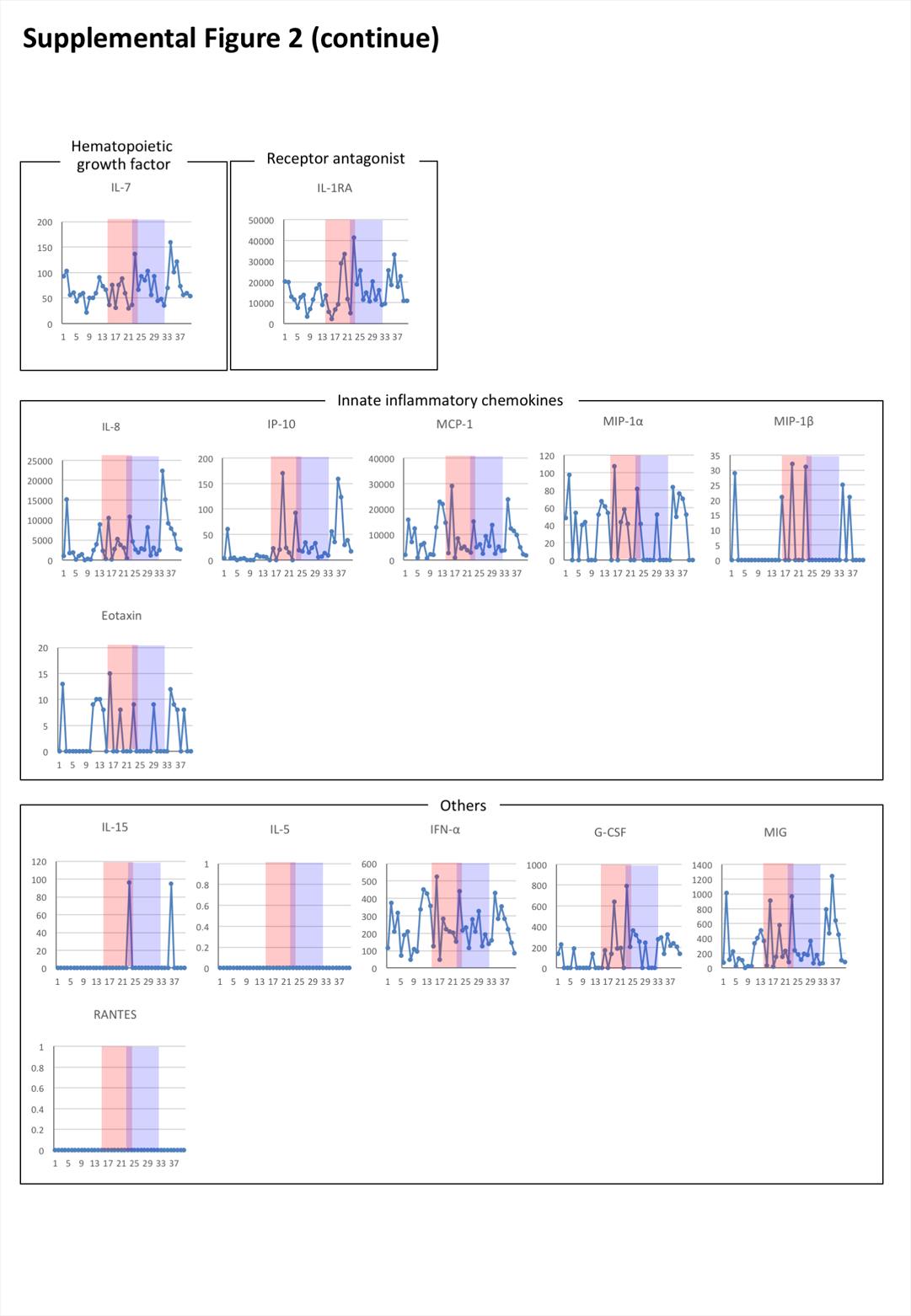

Supplement: Supplementary file 4 — Figure S2. Supporting info item [file CRE2-3-198-s004.tiff]

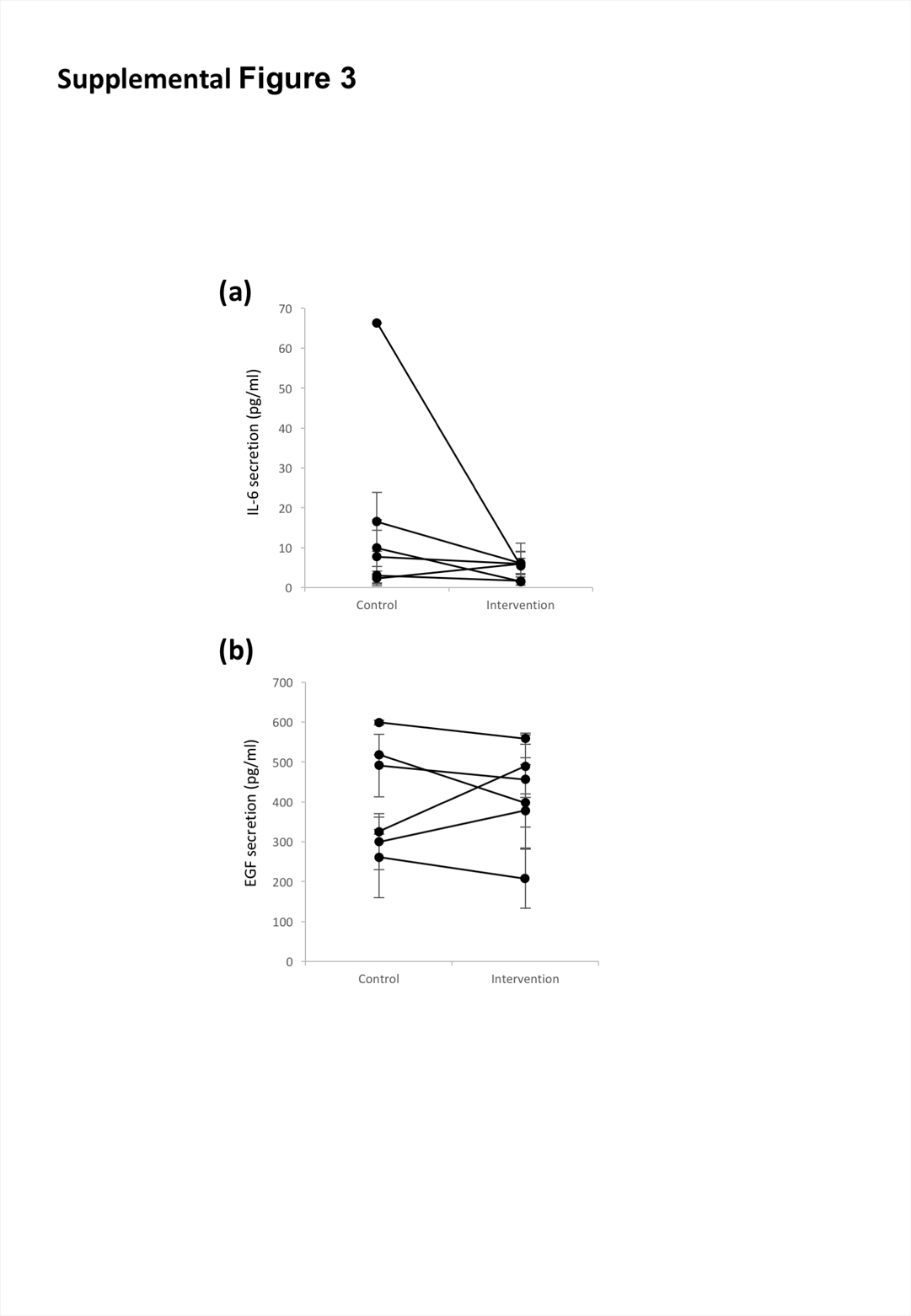

Supplement: Supplementary file 5 — Figure S3. Supporting info item [file CRE2-3-198-s005.tiff]
